# The Effects of Perioperative Corticosteroids on Postoperative Complications After Pancreatoduodenectomy: A Debated Topic of Systematic Review and Meta-analysis

**DOI:** 10.1245/s10434-024-16704-9

**Published:** 2025-01-02

**Authors:** Haonan Liu, Kongyuan Wei, Ruiqi Cao, Jiaoxing Wu, Zhengyuan Feng, Fangzhou Wang, Cancan Zhou, Shuai Wu, Liang Han, Zheng Wang, Qingyong Ma, Zheng Wu

**Affiliations:** 1 Department of Hepatobiliary Surgery, The First Affiliated Hospital of Xi’an Jiaotong University, Xi’an, Shaanxi China; 2 Pancreas Center, Xi’an Jiaotong University, Xi’an, Shaanxi China

**Keywords:** Corticosteroids, pancreatoduodenectomy, Meta-analysis, Complications

## Abstract

**Background:**

The intraoperative administration of corticosteroids has been shown to improve postoperative outcomes in patients undergoing surgery; however, the impact of corticosteroids on complications following pancreatoduodenectomy (PD) remains controversial.

**Objective:**

This study aimed to evaluate the efficacy of perioperative corticosteroids on postoperative complications after PD.

**Materials and Methods:**

A comprehensive search was conducted using the PubMed, Embase, and Web of Science databases for studies published prior to 1 July 2024. Of 7418 articles identified, a total of 5 studies were eligible for inclusion in this meta-analysis. The primary outcome was incidence of postoperative major complications (PMCs), while the additional outcomes were incidences of postoperative pancreatic fistulas (POPFs), infection, delayed gastric emptying (DGE), post-pancreatectomy hemorrhage (PPH), bile leakage, reoperation, and 30-day mortality. The study was registered in the PROSPERO database (CRD42024524936).

**Results:**

Finally, 5 studies involving 1449 patients (537 with corticosteroids and 912 without corticosteroids) were analyzed. Intraoperative corticosteroids were not associated with any improvement in PMCs (*p* = 0.41). The incidence of POPF (*p* = 0.12), infectious complications (*p* = 0.15), or DGE (*p* = 0.81) were not significantly different between the two groups. No obvious differences were found in the incidence of PPH (*p* = 0.42), bile leakage (*p* = 0.68), 30-day mortality (*p* = 0.99), or reoperation (*p* = 0.26).

**Conclusion:**

Perioperative corticosteroids did not significantly demonstrate any protective advantage in terms of postoperative complications after PD. This finding may serve as a reference for the perioperative use of corticosteroids in pancreatic surgery. Well-designed clinical trials are warranted in the near future in order to provide high-level evidence.

**Supplementary Information:**

The online version contains supplementary material available at 10.1245/s10434-024-16704-9.

Pancreatic ductal adenocarcinoma (PDAC) is a highly aggressive disease with a poor prognosis and high mortality rate.^1,2^ Generally, pancreatoduodenectomy (PD) is considered the most effective treatment for tumors located in the head or uncinate process of the pancreas.^3^ Traditional PD involves resection of the head of the pancreas, duodenum, distal common bile duct, and gallbladder, followed by reconstruction to restore the continuity of the digestive tract, making it one of the most challenging and complex surgeries.^4^ PD is frequently associated with a high incidence of adverse events.^3,5,6^ A recently published systematic review of 63,229 PD surgeries reported a postoperative mortality rate of 1.7%, overall complication rate of 54.7%, and severe complication rate of 25.5%. Common postoperative complications, including delayed gastric emptying (DGE; 14.9%), postoperative pancreatic fistulas (POPFs; 14.3%), post-pancreatectomy hemorrhage (PPH; 6.8%), biliary fistula (4.4%), and 30-day mortality (1.7%) not only prolong hospital stays and increase medical costs significantly but also have a substantial impact on patient prognosis.^7^ Furthermore, another study suggested that postoperative complications after PDAC resection were significantly correlated with reduced overall survival.^8^

Dexamethasone, a synthetic glucocorticoid with anti-inflammatory, anti-allergic, and immunosuppressive properties, has been routinely used in clinic for many years.^9^ Perioperative low-dose administration of dexamethasone has been approved as the standard for preventing and treating postoperative nausea and vomiting (PONV);^10–12^ however, concerns arise among surgeons regarding the potential for dexamethasone to increase the risk of anastomotic fistula, delay healing, and consequently lead to postoperative adverse events, as the inflammatory response plays a crucial role in tissue healing.^13–15^ Steroid therapy has also been linked to an elevated risk of postoperative infection.^16^ Consequently, the use of dexamethasone in PD, a complex major abdominal surgery, has not been widely accepted by pancreatic surgeons. Hydrocortisone is also a corticosteroid hormone produced by the cortex of adrenal glands directly involved in stress response. Dexamethasone is a long-acting steroid with a biological half-life of up to 72 h, while hydrocortisone is short-acting (8–12 h); however, 100 mg of hydrocortisone is equivalent to 4 mg of dexamethasone, and the effects of these two drugs are similar.^17^ Laaninen et al.^18^ conducted a randomized controlled trial (RCT) where multiple perioperative doses of hydrocortisone significantly reduced the overall incidence of postoperative complications in patients with PD, especially POPFs and PPH. The authors speculated that this may be attributable to the anti-inflammatory effects of hydrocortisone, when postoperative pancreatic inflammation may be the mediator of complications after PD. Therefore, further research is needed to elucidate the specific role of dexamethasone in postoperative complications after PD.

In recent years, several retrospective studies have evaluated the potential of perioperative corticosteroids in reducing postoperative complications in patients undergoing PD by comparing the short- and long-term outcomes of patients who received corticosteroids with those who did not;^18–22^ however, the findings remain inconclusive. To date, there have been no relevant systematic reviews or meta-analyses on this topic. This study conducted a comprehensive review of the existing literature to assess the specific impact of perioperative corticosteroids on postoperative complications in PD patients.

## Methods and Materials

### Search Strategies

We performed a systematic literature search of the PubMed, Embase, and Web of Science databases for studies published before 1 July 2024 (including cohort and case-control studies) that were related to perioperative corticosteroids, using a combination of the following terms: ‘dexamethasone [title/abstract]’ or ‘hydrocortisone [title/abstract]’ and ‘pancreas*[title/abstract]’. To evaluate the inclusion of other potential articles, references of selected articles were also screened. The Preferred Reporting Items for Systematic Reviews and Meta-Analyses (PRISMA) guidelines were followed when retrieving the articles.^23^ Detailed information on the search strategies are provided in electronic supplementary material (ESM) Table 1. The concrete protocol of this study is available in the PROSPERO database (identifier CRD42024524936).

### Study Selection

Each study was independently assessed by two reviewers (Haonan Liu, Kongyuan Wei), in accordance with predefined eligibility criteria. Any disagreements were resolved in consultation with a third reviewer (Zheng Wu) in necessity. The following inclusion criteria were used: (1) research including humans; (2) RCTs and retrospective studies comparing corticosteroids with non-corticosteroids (including cohort and case-control studies); and (3) when duplicate publications were encountered, only the most updated and complete reports were included. All meetings, abstracts, letters, expert opinions, case reports, reviews, and articles not in English were excluded, along with articles for which we could not obtain data. The screening process, according to the PRISMA flowchart,^24^ is reported in ESM Table 2.

### Data Extraction

Two researchers independently collected data using a standardized data abstraction form. Data on study and patient characteristics, primary outcomes, and other outcomes were collected. Unless otherwise specified, our meta-analysis used standard deviations and mean values. If no standard deviation or mean was reported, the median or quartile distance was calculated as the mean or standard deviation, respectively. Data were extracted as follows: author, year of publication, study design, study period, number of patients, age, sex, body mass index (BMI), primary outcome (postoperative major complications [PMCs]; Clavien–Dindo grade ≥III),^25^ and additional outcomes (POPFs, infection, DGE, PPH, bile leakage, reoperation, and 30-day mortality). POPFs, DGE, and PPH were defined according to the International Pancreatic Surgery Research Group (ISGPS).^26,27^ In all the reviewed literature, POPF types B and C are confirmed. The previously designated ‘POPF A’ has now been redefined as a ‘biochemical leak’ and is no longer referred to as a true pancreatic fistula. All surgical complications were graded using the Clavien–Dindo classification system.^28^ The grading criteria of POPFs and PMCs for each article are presented in Table 1.

**Table 1:** Characteristics and quality of the included studies

References	Year published	Design	Country	Study period	Intervention	No. of participants		Age, years		Sex, males (%)		BMI, kg/m^2^		POPF (updated ISGPS definition)	PMCs (Clavien–Dindo ≥III)	Study quality/ risk of bias
						Experimental	Control	Experimental	Control	Experimental	Control	Experimental	Control			
Laaninen et al.^18^	2016	Single-center, double-blind, RCT	Finland	February 2011–May 2015	Hydrocortisone	28	34	63 (43–82)	66 (45–83)	18 (64.3)	21 (62)	25 (18–38)	26 (17–38)	Yes	Yes	Low
Chen et al.^19^	2024	Multicenter, double-blind, RCT	China	October 2022–September 2023	Dexamethasone	134	131	61.7 ± 9.9	62.6 ± 11.1	82 (61.2)	78 (59.5)	22.7 ± 3.2	23.0 ± 3.0	Yes	Yes	Low
Newhook at al.^20^	2021	Retrospective study	America	July 2011–October 2018	Dexamethasone	223	150	65 (40–85)	65.5 (35–88)	87 (51.9)	81 (48.1)	26.54 (16.9–46.8)	27.07 (17.9–39.6)	Yes	Yes	Moderate
Sandini et al.^21^	2018	Retrospective study	America	January 2007–December 2015	Dexamethasone	562	117	65 (55–71)	67 (60–75)	38 (32.5)	284 (50.5)	25.9 (22.8–27.6)	27.0 (24.2–28.8)	Yes	Yes	Moderate
Kant et al.^22^	2024	Single-center, three-arm, triple-blinded, RCT	India	June 2018–June 2020	Hydrocortisone	35	35	52 (43–65)	59 (49–61)	20 (57.1)	27 (77.1)	–	–	Yes	Yes	Low

*RCT* randomized controlled trial, *BMI* body mass index, *POPF* postoperative pancreatic fistula, *ISGPS* International Study Group of Pancreatic Surgery, *PMCs* postoperative major complications

### Qualitative Assessment

Our research assessed the risk of bias in the included non-randomized studies according to the Risk of Bias in Non-randomized Studies of Interventions (ROBINS-I) tool.^29^ For each study, the overall risk of bias was determined based on risk judgments in the seven domains. Risk-of-bias appraisal in the included RCTs was carried out using the second version of the Cochrane Collaboration’s Tool for Assessing Risk of Bias in Clinical Trials (RoB-2 tool).^30^ In each study, two reviewers evaluated the risk of bias independently and resolved conflicts through discussion between the two reviewers.

### Data Synthesis and Analysis

All statistical analyses were performed using Review Manager 5.4 software (The Cochrane Collaboration, Oxford, UK). Weighted mean differences and 95% confidence intervals (CIs) were calculated based on the inverse variance method for continuous outcomes. The study expressed dichotomous variables as odds ratios (ORs) along with their corresponding 95% CIs, which were calculated using the Mantel–Haenszel (M-H) method. Statistical heterogeneity was assessed using the *I*-square test (*I*^2^) and was interpreted as follows: *I*^2^* ≥* 50% indicated high heterogeneity, and the random-effects model was applied, otherwise the fixed-effects model was used.^29^ Data were also presented descriptively if inappropriate for meta-analysis. Potential publication bias of the results was assessed using Egger’s funnel plots.^30^ In addition, our research conducted a ‘leave-one-out’ sensitivity analysis to identify the cause of heterogeneity. Statistical significance was defined as *p* < 0.05, and a 95% CI was calculated for efficiency measures.

## Results

### Search Results

**Fig. 1:**
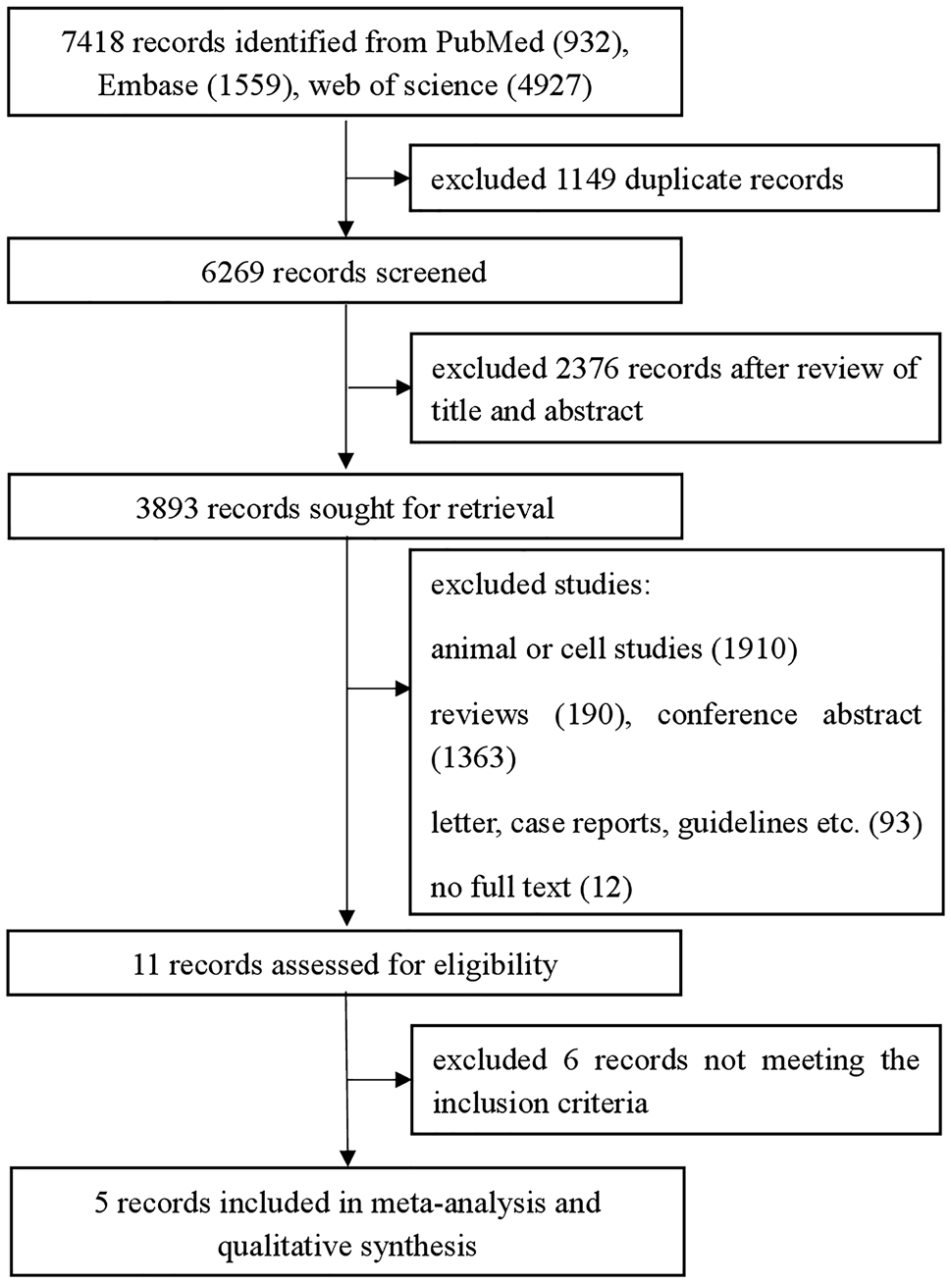
Overview of the literature search and selection process

The initial search identified 7418 articles, of which 6269 were retained after the removal of 1149 duplicates. After reviewing the titles and abstracts, 2376 articles were excluded, along with another 3882 articles that were excluded based on the defined exclusion criteria (reviews, letters, animal studies, guidelines, non-English-language articles, case reports, and studies without control groups). We conducted full-text reviews of 11 articles to assess compliance with the inclusion criteria. Finally, five studies fulfilled all inclusion criteria and were included in this meta-analysis (Fig. 1).

### Characteristics of the Included Studies

Table 1 describes the study and patient baseline characteristics. Three RCTs^18,19,22^ and two prospectively maintained pancreatic surgery database reviews^20,21^ that met the inclusion criteria were included in our systematic review and meta-analysis. Five studies including a total of 1449 patients reported all relevant postoperative outcomes. The glucocorticoids administered in the studies included hydrocortisone or dexamethasone. Specifically, three studies^19–21^ involved a single dose of dexamethasone in a dose range of 4–10 mg, and two studies^18,22^ used a hydrocortisone regimen consisting of 100 mg intravenously, administered three times daily (every 8 h) for 2 days.

### Quality Assessment of the Included Studies

The literature included in this study included three RCTs and two non-RCTs. Quality assessment of the RCTs was conducted using the RoB-2 tool and was graded as low-risk.^18,19,22^ The two non-RCT studies evaluated using the ROBINS-I tool were both rated as moderate risk^20,21^ (Table 1).

### Meta-analysis

#### Primary Outcomes

**Fig. 2:**
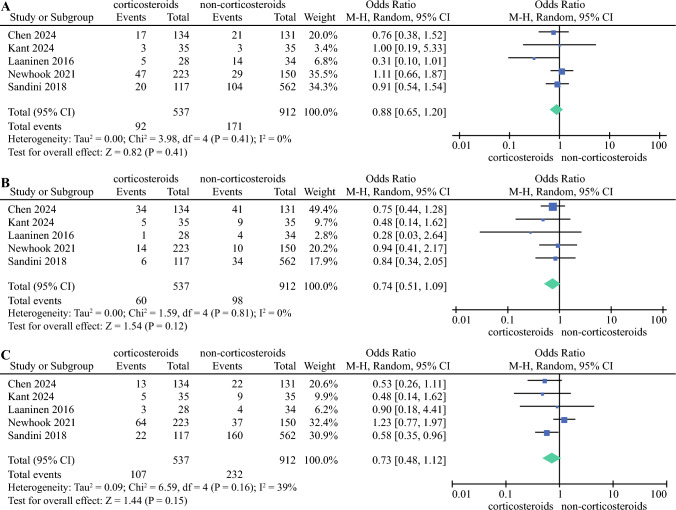
Forest plot showing the association of perioperative corticosteroids with **A** postoperative major complications; **B** postoperative pancreatic fistulas; and **C** infectious complications after the surgery. *M-H* Mantel-Haenszel, *CI* confidence interval, *df* degrees of freedom

The incidence of PMCs was reported in all studies^18–22^ (1449 participants). In three studies,^18,19,21^ patients who received intraoperative corticosteroid injections had a reduced risk of PMCs compared with those who did not, however this was not statistically significant across all studies (OR 0.88, 95% CI 0.65–1.20; *p* > 0.05). There was low heterogeneity in the pooled results (*I*^2^ = 0%, *p* = 0.41). The results of the meta-analysis are shown in Fig. 2a.

#### Additional Outcomes

**Fig. 3:**
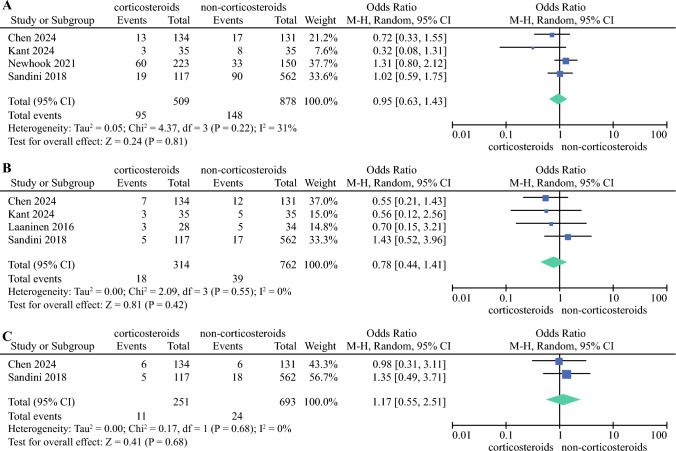
Forest plot showing the association of perioperative corticosteroids with **A** delayed gastric emptying; **B** post-pancreatectomy hemorrhage; and **C** bile leakage after surgery. *M-H* Mantel-Haenszel, *CI* confidence interval, *df* degrees of freedom

**Fig. 4:**
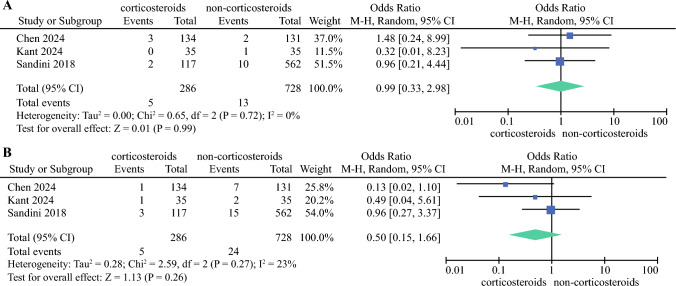
Forest plot showing the association of perioperative corticosteroids with **A** 30-day mortality; and **B** reoperation after surgery. *M-H* Mantel-Haenszel, *CI* confidence interval, *df* degrees of freedom

Five studies^18–22^ (1449 patients) reported POPFs (Fig. 2b), and in all studies, patients in the corticosteroid group showed a lower incidence of POPFs but there was no significant difference (OR 0.74, 95% CI 0.51–1.09; *p* = 0.12). The results were homogeneous (*I*^2^ = 0%, *p* = 0.81). The ‘infectious’ complication was reported in five studies^18–22^ (1449 patients) (Fig. 2c), and the results showed there was no significant change in infection between the two groups (OR 0.73, 95% CI 0.48–1.12; *p* > 0.05). Pooled results were heterogeneous (*I*^2^ = 39%, *p* = 0.16). DGE was reported in four studies^19–22^ (1387 patients) (Fig. 3a), however the groups did not differ significantly (OR 0.95, 95% CI 0.63–1.43; *p* > 0.05). The results were heterogeneous (*I*^2^ = 31%, *p* = 0.22). PPH was also reported in four studies^18,19,21,22^ (1076 patients) (Fig. 3b) and there was no significant difference (OR 0.78, 95% CI 0.44–1.41; *p* > 0.05). Pooled results were homogeneous (*I*^2^ = 0%, *p* = 0.55). Two studies^19,21^ (944 patients) (Fig. 3c) reported bile leakage, however there was no significant difference (OR 1.17, 95% CI 0.55–2.51; *p* = 0.68). In addition, 30-day mortality and reoperation were reported in three studies^19,21,22^ (1014 patients). There were also no significant differences in the incidence of 30-day mortality (OR 0.99, 95% CI 0.33–2.98; *p* > 0.05) and reoperation (OR 0.50, 95% CI 0.15–1.66; *p* > 0.05) between the two groups (Fig. 4a, b).

### Sensitivity Analysis

Sensitivity analysis was performed for the moderately heterogeneous outcomes (postoperative infection and DGE) using a one-by-one exclusion method to determine the stability of the results. After removing some of the studies, the combined effect size remained similar, indicating that the results of this study were relatively stable.

### Publication Bias

Funnel plot revealed that the studies were evenly distributed on both sides, and the distribution was approximately symmetrical, suggesting no significant publication bias.

## Discussion

With the improvement of operation procedures, the mortality rate after PD has decreased to 3–5%;^31^ however, the associated postoperative complications are still as high as 50%.^32^ According to the available literature, the results demonstrated that the use of corticosteroids in PD had no impact on the risk of postoperative complications. This indicated that corticosteroids cannot be applied in the perioperative period to prevent postoperative complications of PD on account of existing results, but it may provide some reference for the prevention of PONV after pancreatic surgery.

Several studies have previously shown that the use of corticosteroids in PD can improve long-term survival outcomes;^1,21,33^ however, there are conflicting conclusions regarding whether corticosteroids can reduce postoperative complications. There are no meta-analyses addressing this controversial issue. In a Finnish trial, Laaninen et al.^18^ conducted an RCT that included 155 patients scheduled for PD, in which patients were treated with hydrocortisone 100 mg (in total, eight doses every 8 h) or placebo. The RCT suggested that in high-risk patients, overall post-PD complications can be significantly reduced with hydrocortisone treatment (18% vs. 41%; *p* < 0.05; Clavien–Dindo grade III–IV). Sandini et al.^21^ analyzed 679 PDs from a prospectively maintained database and certified that a single intraoperative dose of dexamethasone was relevant to the improvement of median overall survival (46 vs. 22 months; *p* = 0.017); however, they failed to demonstrate any protective advantage in terms of morbidity after PD, and only showed an association with a decrease in infectious complications (18.8% vs. 28.5%; *p* = 0.032). Newhook et al.^20^ retrospectively abstracted 298 patients who underwent PD and concluded that dexamethasone played no role in mitigating short-term PMCs (21.1 vs. 19.3%; *p* = 0.68), POPFs (6.3 vs. 6.7%; *p* = 0.88), and infectious complications (28.7 vs. 24.7%; *p* = 0.39), or to confer any long-term survival benefit. Chen et al.^19^ conducted a multicenter, double-blind RCT involving 254 patients with PD, concluding that the incidence of PMCs (12.7% vs. 16.0%; *p* = 0.439) and POPFs (25.4% vs. 31.3%; *p* = 0.286) was not significantly different between the two groups. Kant et al.^22^ carried out a triple arm, randomized, placebo-controlled trial that included 70 participants with PD and found that overall complications were significantly lower in the hydrocortisone group than in the placebo group (16 [45.7%] vs. 28 [80.0%]; *p* = 0.006). Other complications were comparable between the two groups. Although some previous studies have shown that the use of corticosteroids in PD can significantly reduce the incidence of some postoperative complications, our systematic review did not reach such a conclusion.

Previous studies have shown that perioperative corticosteroids can reduce the incidence of some postoperative complications and length of hospital stay after major abdominal surgery, but not after pancreatic surgery. Blank et al.^34^ demonstrated that in solid cancer resection, corticosteroids were associated with decreased 1-year mortality and a reduced risk of cancer recurrence in patients not responding to checkpoint inhibitor therapy. Moreover, corticosteroids increased the risk of postoperative hyperglycemia, but no benefits in surgical site infections were observed. Regarding the effects of gastrointestinal surgery on intestinal function, McKechnie et al.^35^ published a meta-analysis in 2023 that included seven studies assessing the postoperative impacts of dexamethasone in colorectal surgery. This meta-analysis showed that perioperative intravenous dexamethasone may reduce prolonged postoperative ileus but there was no significant effect on postoperative infection complications. Zhang et al.^36^ also showed that a single 8 mg dose of intravenous dexamethasone administered during surgery for inflammatory bowel disease decreased the incidence of prolonged postoperative ileus and postoperative pain intensity, while also shortening the postoperative length of stay. There were no significant differences in postoperative nausea or vomiting, major postoperative complications, or surgical site infections between the groups. A single-center, two-arm, parallel RCT^37^ involving 126 patients who underwent open or laparoscopic bowel surgery found that a single 8 mg dose of intravenous dexamethasone significantly reduced abdominal distension and improved gastrointestinal tolerance to food. Furthermore, Asehnoune et al.^38^ conducted a randomized, double-blind, placebo controlled trial of 1222 adults requiring major non-cardiac surgery in which dexamethasone was not found to reduce the incidence of complications and death in patients 14 days after surgery. A multicenter RCT^39^ on colorectal, pancreatic, or liver surgery in which dexamethasone (16 mg) and 20% albumin (100 mL) were administered to patients, indicated that these patients experienced fewer postoperative complications than in the control group. Steinthorsdottir et al.^40^ conducted a single-center, double-blind, parallel-group RCT investigating the preoperative treatments of methylprednisolone 10 mg/kg versus dexamethasone 8 mg in patients scheduled for open liver resection. That study involved 174 patients and concluded that a high dose of preoperative glucocorticoids did not reduce acute postoperative complications after open liver resection. Aldrighetti et al.^41^ conducted a prospective randomized study comparing a steroid group with a placebo group. Preoperative steroid administration had a significant protective effect on ischemia-reperfusion injury and postoperative complications in that study.

Although these studies were highly heterogeneous, including many types of surgeries and patients with different baseline risk profiles, all studies illustrated the important role that perioperative corticosteroids can play in other procedures.

The appearance observed in our study did not have a positive effect on postoperative complications of PD, which is inconsistent with the conclusions of other abdominal surgeries, mainly owing to the complexity and specificity of pancreatic surgery. PD is considered to be one of the most challenging surgeries due to its complex operation, high risk, and high postoperative morbidity and mortality associated with postoperative complications such as PPH and POPFs.^42,43^ PD involves the removal and reconstruction of multiple organs of the digestive system, which is difficult, time-consuming, and highly invasive, and can cause cascading inflammatory reactions.^44^ At the same time, the pancreas is a secretory organ, thus changes in anatomical structure after PD will lead to the obstruction of biliary pancreatic secretion, change in pH, and inactivation of pancreatic enzymes.^45^ In addition, due to the special pathophysiological changes of pancreaticoduodenal diseases, most patients have nutrition-related problems before surgery and are more prone to complications after surgery.^46^ Surgical traumatic stress, digestive tract reconstruction, and postoperative glandular dysfunction will affect the metabolic and nutritional status of the body to varying degrees, causing a variety of serious complications such as POPF, thus affecting the postoperative recovery and clinical outcome of patients. Therefore, compared with other abdominal operations, perioperative physiological changes caused by stress, fluid displacement and blood loss are more significant in PD patients. It is possible that this is the reason for the insignificant results in pancreatic resection. However, the mechanism of perioperative corticosteroids on postoperative complications after major pancreatic surgery remains to be fully elucidated. Cell experiments have confirmed that dexamethasone can inhibit cell migration and invasion by inhibiting transcription factors of epithelial-mesenchymal transformation of colon cancer cells under hypoxia conditions, and can also restore the normal phenotype of colon cancer cells under normoxic conditions.^47^ PDAC can also develop with an insufficient blood supply, and its cell microenvironment is similar to that of colon cancer; however, it remains unclear whether the same mechanisms of action can play an important clinical role in pancreatic cancer. In addition, animal studies verified that inflammation increases the metastasis of pancreatic cancer cells, whereas dexamethasone can abolish this biological behavior.^48^ Therefore, the role of corticosteroids in PD surgery warrants further exploration through large-scale trials.

In this study, we acknowledge that there are some limitations. On the one hand, our study included only five existing pieces of literature, of which only three were RCT studies carried out in high-volume surgical centers, thus leading to bias. The participants in these five studies came from different countries and regions, i.e. one from China and the remaining four from other countries. There were also some differences in the inclusion and exclusion criteria among the five studies. For instance, although PD was performed in all studies, the underlying diseases of the included groups were different. Therefore, it is not clear whether dexamethasone has a positive effect on postoperative complications in specific groups. On the other hand, the corticosteroids in our study included dexamethasone and hydrocortisone. The anti-inflammatory efficacy achieved by the two regimens is theoretically similar. However, no studies have reported the effects of a single injection of long-acting dexamethasone and multiple injections of short-acting hydrocortisone in the context of pancreatic surgery. Moreover, the administration methods of the five studies were also inconsistent. Furthermore, corticosteroids act as anti-inflammatory agents to reduce excessive inflammation after surgery and have been found to be associated with lower C-reactive protein levels.^18^ Studies have shown that increased levels of postoperative serum C-reactive protein are associated with an elevated incidence of postoperative pancreatic complications.^49–51^ Nonetheless, our meta-analysis did not include postoperative C-reactive protein as an indicator, which prevents us from explaining our results from this perspective and understanding the specific mechanisms of our findings. Nevertheless, the research agrees with previous trials that the use of corticosteroids in PD does not increase the incidence of postoperative complications. Large-scale RCTs with higher evidence levels are needed for verification.

## Conclusion

This study presents the first meta-analysis comparing corticosteroids and non-corticosteroids in PD. We concluded that perioperative corticosteroids did not significantly demonstrate any distinction in terms of postoperative complications after PD, including PMCs, POPFs, infectious complications, DGE, PPH, bile leakage, reoperation, and 30-day mortality. This may serve as a reference for the perioperative application of corticosteroids in PD. Well-designed clinical trials are warranted in the near future to provide high-level evidence regarding the potential benefits of corticosteroids for the targeted population undergoing pancreatic surgery. Furthermore, the specific mechanisms of the effect of corticosteroids on the postoperative complications of PD still needs to be explored.

## Supplementary Information

Below is the link to the electronic supplementary material.

Supplementary file1 (DOCX 18 kb)

Supplementary file2 (DOCX 31 kb)

Supplementary file3 (TIF 181 KB)
