# Supplementary material for: The Effects of Perioperative Corticosteroids on Postoperative Complications After Pancreatoduodenectomy: A Debated Topic of Systematic Review and Meta-analysis
Source: Ann Surg Oncol. 2025 Jan 2;32(4):2841–51. doi: 10.1245/s10434-024-16704-9 (PMC11882649; doi:10.1245/s10434-024-16704-9)
Supplement: Supplementary file 1 — Supplementary file1 (DOCX 18 kb) [file 10434_2024_16704_MOESM1_ESM.docx]

**Supplementary Table 1.** Search strategies.

| **PubMed** | |
| --- | --- |
| #3 | #1 AND #2 |
| #2 | " pancreas*" [title/abstract] |
| #1 | " dexamethasone" or " hydrocortison" [title/abstract] |
| **Embase** | |
| #3 | #1 AND #2 |
| #2 | ‘pancreas*’ |
| #1 | ‘dexamethasone’ or ‘hydrocortisone’ |
| **Web of Science** | |
| #1 | TS= ("dexamethasone” or " hydrocortison") |
| #2 | TS= ("pancreas*") |
| #3 | #1 AND #2 |
