# Supplementary figures and images for: The Effects of Perioperative Corticosteroids on Postoperative Complications After Pancreatoduodenectomy: A Debated Topic of Systematic Review and Meta-analysis
Source: Ann Surg Oncol. 2025 Jan 2;32(4):2841–51. doi: 10.1245/s10434-024-16704-9 (PMC11882649; doi:10.1245/s10434-024-16704-9)

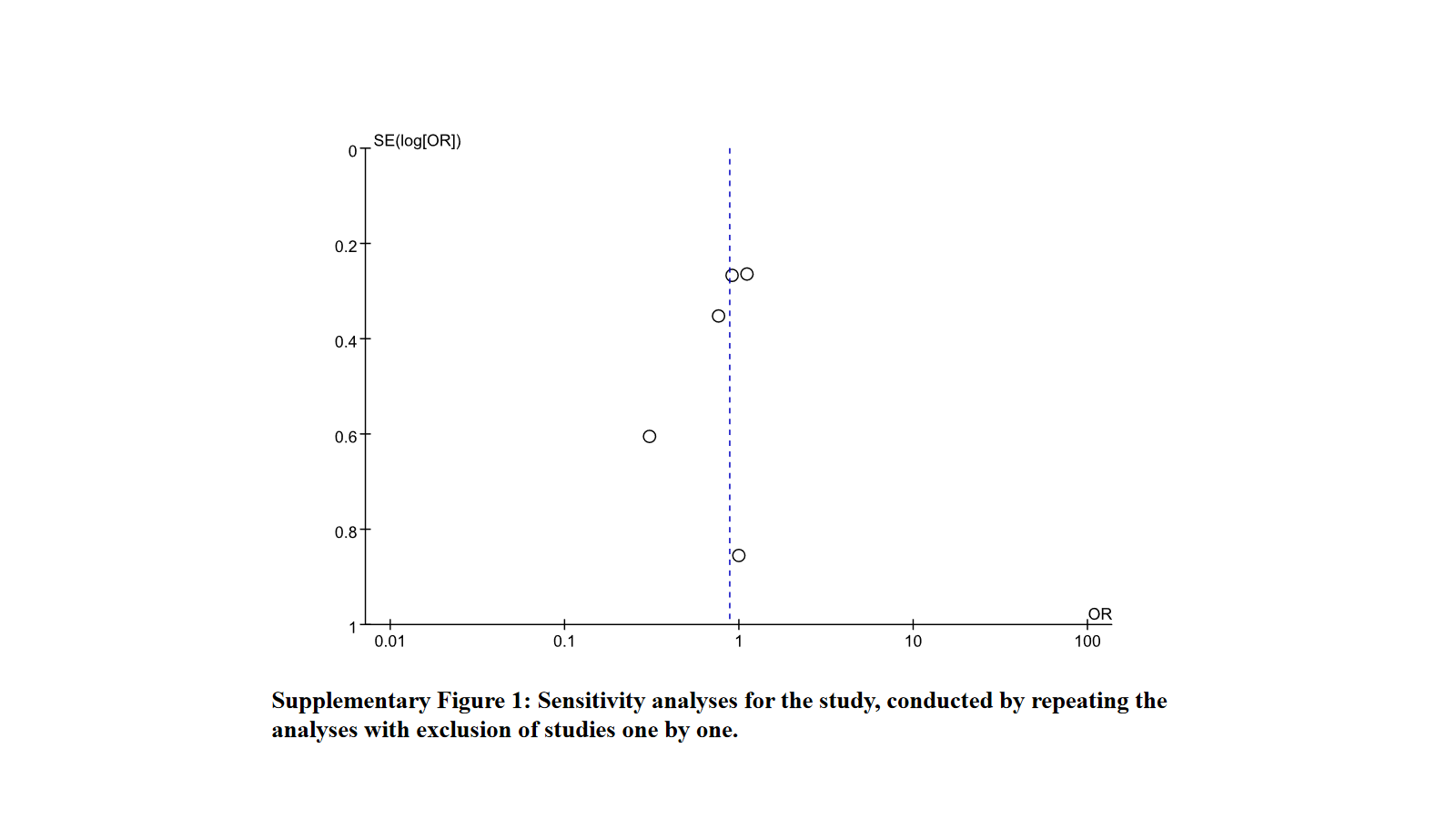

Supplement: Supplementary file 3 — Supplementary file3 (TIF 181 KB) [file 10434_2024_16704_MOESM3_ESM.tif]
